# Pharmacotherapy Agents in Lymphedema Treatment: A Systematic Review

**DOI:** 10.7759/cureus.6300

**Published:** 2019-12-05

**Authors:** Antonio J Forte, Daniel Boczar, Maria T Huayllani, Xiaona Lu, Sarah A McLaughlin

**Affiliations:** 1 Plastic Surgery, Mayo Clinic Florida Robert D. and Patricia E. Kern Center for the Science of Health Care Delivery, Jacksonville, USA; 2 Plastic Surgery, Yale University, New Haven, USA; 3 Surgery, Mayo Clinic Florida Robert D. and Patricia E. Kern Center for the Science of Health Care Delivery, Jacksonville, USA

**Keywords:** lymphedema, breast cancer lymphedema, pharmacotherapy, plastic surgery, individualized medicine, targeted therapy, pharmacologic treatment, inflammation, upper extremity, lower extremity

## Abstract

It is estimated that one in every six patients undergoing solid cancer treatment will develop secondary lymphedema. We conducted a systematic review of publications assessing the potential use of pharmacotherapy agents in lymphedema treatment. The search was conducted on PubMed and eligibility criteria excluded papers that investigated other therapies or focused on primary lymphedema. From 285 potential papers found in the literature, seven studies fulfilled the eligibility criteria. Different types of therapies were proposed, but all of them interfered with inflammation in the lymphedema tissue. Interestingly, the majority of publications were clinical, and three authors conducted randomized, placebo-controlled, double-blinded clinical studies. Promising results were observed for the oral administration of ketoprofen or selenium and topical tacrolimus. Pharmacotherapy agents were successfully described in lymphedema treatment in clinical and experimental studies. The benefits of delivering ketoprofen, selenium, or tacrolimus in lymphedema were noticed, and these therapies were easily delivered and well-tolerated.

## Introduction and background

It is estimated that one in every six patients with solid cancer will develop lymphedema. In the United States alone, five to six million people are affected [1]. It is well-accepted by the scientific community that fibrosis and inflammation play a primary role in lymphedema physiopathology [2-4]. While searching for therapies to alleviate or potentially cure lymphedema, authors have proposed the utilization of agents that modulate tissue inflammation, fibrosis, and lymphangiogenesis. However, to date, most studies have proposed the utilization of lymphangiogenic growth factors, such as vascular endothelial growth factor C (VEGF-C), which could potentially increase the risk of metastasis [5-6].

Outcomes in lymphedema treatment are still unpredictable, bringing attention to the investigation of new therapies that could be easily and effectively translated to patients [7]. We conducted a systematic review of literature on the use of pharmacotherapy agents in lymphedema treatment.

## Review

Material and Methods

Search Strategy

On August 2, 2019, two reviewers (D.B. and M.T.H.) conducted independent searches using the PubMed database without timeframe limitations, initially through the title and abstract screen and then by a full-text review. Disagreements regarding article identification and final selection for the inclusion of literature were resolved by another reviewer (A.J.F.). The search was done using the following keywords: (((((((("anti-inflammatory agents") OR anti-inflammatory) OR Ketoprofen) OR ("Leukotriene B4 antagonists and inhibitors")) OR "Leukotriene B4") OR NSAID)) OR (("Immunosuppressive agents") OR Tacrolimus)) AND ((lymphedema) OR breast cancer lymphedema). Bibliographies of studies that fulfilled the study eligibility criteria were also examined, looking for articles not present in our initial search. This study followed the guidelines outlined in the Preferred Reporting Items for Systematic reviews and Meta-Analyses (PRISMA).

Selection Criteria

Eligibility criteria included studies reporting data on the potential use of pharmacologic agents as therapy in the treatment of cancer-related secondary lymphedema. Therefore, we excluded papers addressing other topics (eg, lymphedema physiopathology, anesthetic or surgical procedures, or the treatment of the inflammatory complications of lymphedema) and pharmacotherapy applied to other causes of lymphedema (eg, idiopathic, infectious, or rheumatologic). Abstracts, presentations, reviews, meta-analyses, and non-English language publications were also excluded.

Data Extraction and Processing

Extracted data included the year of study, country, type of study, the model used for experiments, class of medication, name of the drug, and therapy delivery. Data extraction from articles, tables, and figures was performed by two reviewers (D.B. and M.T.H.), with the accuracy of data entry confirmed by an additional reviewer (A.J.F.).

Results

Description of Studies

**Figure 1:**
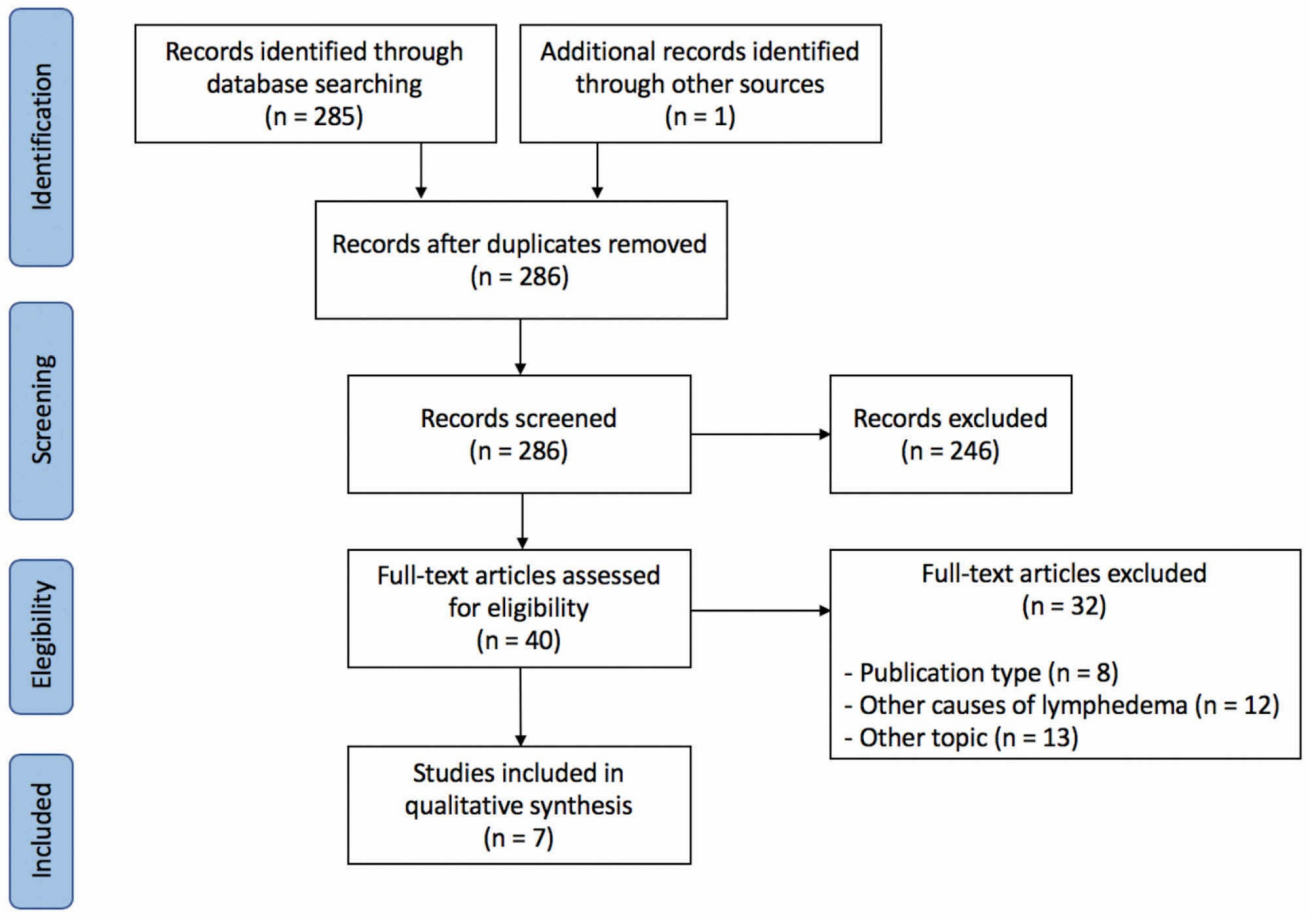
PRISMA Flow Chart of Included Studies in the Systematic Review of the Literature. PRISMA: Preferred Reporting Items for Systematic reviews and Meta-Analyses

From 286 potential papers found in the literature, seven studies fulfilled the study eligibility criteria (Figure 1; Table 1). The potential use of pharmacotherapy as a therapy in lymphedema treatment was described in studies from numerous countries. The first publication on the topic was a report on the injection of cyclophosphamide in four lymphedema patients. The utilization of different pharmacotherapy agents was proposed to treat lymphedema, but all of them involved agents with the capacity to control inflammation. Interestingly, the majority of studies (5 of 7) were clinical. The remaining publications were experimental studies in lymphedema models induced on mice.

**Table 1:** Summary of Studies Investigating Pharamacotherapy Agents in Lymphedema Treatment Abbreviations: UK, United Kingdom;

Author	Year	Country	Study type	Model/Cases	Name	Delivery
Kitchen et al. [8]	1971	UK	Case series	4 patients	Cyclophosphamide	Injection
Kasseroller et al. [9]	2000	Austria	Placebo-controlled, double-blind	179 patients	Sodium Selenite	Oral
Micke et al. [10]	2003	Germany	Case series	48 patients	Sodium Selenite	Oral
Zimmermann et al. [11]	2005	Germany	Placebo-controlled, double-blind	20 Patients	Sodium Selenite	Oral
Nakamura et al. [12]	2009	USA	Experimental	Mice	Ketoprofen, or Pegsunercept (soluble TNF-a receptor R1)	Subcutaneous injection
Gardenier et al. [13]	2016	USA	Experimental	Mice	Tacrolimus	Topical
Rockson et al. [14]	2018	USA	Placebo-controlled, double-blind	55 Patients	Ketoprofen	Oral

Ketoprofen

Nakamura et al. conducted an experimental study on a model of acquired lymphedema on mice, assessing the potential of subcutaneous injections of ketoprofen [12]. They demonstrated that ketoprofen injections reduced tail edema, normalized histologic changes, ameliorated dilated microlymphatics, and reduced epidermal thickness compared to untreated mice. They pointed out that ketoprofen paradoxically increased tumor necrosis factor (TNF)-α expression and cytokine levels. The authors postulated that ketoprofen likely promotes lymphangiogenesis by VEGF C induced through TNF-α. Rockson et al. conducted a clinical study on patients with primary or secondary lymphedema who took oral doses of ketoprofen three times daily for four months [14]. Initially, the study was an exploratory, open-label trial, with 21 patients, in which they noticed considerable lymphedema improvement at four months compared to patients at baseline before treatment. Driven by the positive results, they performed the second phase of the study, as a placebo-controlled clinical trial, in which they recruited 34 patients (16 were treated with ketoprofen and 18 received placebo). Ketoprofen decreased skin thickness and expression of plasma granulocyte colony-stimulating factor (G-CSF) and improved histopathology composite measures [14].

Soluble TNF-α Receptor R1

Nakamura et al. also assessed the subcutaneous injection of pegsunercept, a soluble TNF-α receptor 1 inhibitor [12]. Contrary to the positive results found for ketoprofen, a pegsunercept injection did not result in an improvement of lymphedema in mice as compared to controls. Interestingly, epidermal thickness increased and TNF-α gene expression decreased.

Selenium

Kasseroller and Schrauzer conducted a placebo-controlled, double-blind clinical study in which they recruited 179 patients with breast cancer-related lymphedema (90 treated with sodium selenite and 89 with placebo) who were followed for three months [9]. They pointed out the potential of sodium selenite to reduce lymphedema volume as compared to the placebo. Moreover, it reduced the incidence of erysipelas and improved skinfold index and mobility. Micke et al. conducted a clinical study describing a series of 48 patients with lymphedema (12 on arms and 36 on head and neck) who received daily oral doses of sodium selenite for four to six weeks [10]. They observed circumference reduction and improved the skinfold index on 10 of 12 patients with arm lymphedema. However, differences were not statistically significant, which they attributed to the small sample size. The authors pointed out that treatment with sodium selenite was easily delivered and well-tolerated by their patients [10]. Zimmermann et al. conducted a prospective, randomized, placebo-controlled, double-blind clinical study on 20 patients with lymphedema. They administered sodium selenite pre-, intra-, and postoperative for the treatment of squamous cell carcinoma of the head and neck (10 patients were treated with sodium selenite and 10 received placebo). Compared to the placebo, patients treated with sodium selenite had a statistically significant volume reduction [11].

Tacrolimus

Gardenier et al. conducted an experimental study on mice, applying tacrolimus topically [13]. They noticed that the immunosuppressive potential of this drug affecting T-cells was effective in not only treating established lymphedema but also in preventing the initial development of lymphedema. Treated mice presented with decreased tissue swelling, fibrosis, and T-cell infiltration and increased lymphangiogenesis. Moreover, the authors noticed that treated animals also had improved lymphatic function and decreased dermal backflow [13].

Cyclophosphamide

Kitchen and Garrett published a series of cases describing the injection of cyclophosphamide in four patients with secondary lymphedema due to lymphatic metastasis from breast carcinoma [8]. Injections were done at an outpatient clinic, and no complications were noticed. Two patients presented with clinical improvement in edema and symptom relief [8].

Discussion

To our knowledge, this is the first systematic literature review summarizing studies about the use of pharmacotherapy agents in lymphedema treatment. Different drugs that act by immunologic modulation were proposed. The rationale is that lymphangiogenesis could be potentiated by removing the deleterious manifestations of inflammation. Three authors conducted double-blinded, placebo-controlled studies [9,11,14]. Promising results were demonstrated for the delivery of ketoprofen, selenium, and tacrolimus.

Clinical and experimental studies demonstrated an improvement in lymphedema through the delivery of ketoprofen [12,14]. The idea of using ketoprofen in lymphedema treatment was originated by its capacity to inhibit cyclooxygenase, reducing tissue inflammation [15]. The delivery of selenium was assessed in three publications, all of which demonstrated positive results [9-11]. Although no adverse effects were noted among their patients, the common adverse effects of selenium described in the literature are gastrointestinal symptoms (eg, nausea, vomiting, and diarrhea) and irritation of the respiratory system.

Topical tacrolimus was investigated in one experimental study, leveraging the well-established safety and tolerability of this drug [13]. An advantage of topical delivery is the fact that it decreases the chance of systemic complications. Moreover, topical tacrolimus already has US Food and Drug Administration (FDA) approval for other chronic skin conditions, which is an important point to consider in terms of translating new therapies into the clinical setting. Because the drug would be applied to a large surface area, the authors acknowledged the importance of additional studies to optimize this treatment [13].

We do recognize the presence of several limitations to our study, including the potential for bias in interpreting data collected from other studies. Moreover, we only included papers published in English in this review. The search was conducted only on PubMed and our selection criteria resulted in a small number of included studies. However, we believe that this systematic review summarized valuable data regarding the potential use of pharmacotherapy agents in lymphedema treatment, which can guide future studies to advance the field.

## Conclusions

Studies on the use of pharmacotherapy agents in lymphedema treatment investigated anti-inflammatory drugs. Promising outcomes were reported for the utilization of ketoprofen, selenium, and tacrolimus. Those agents were easily delivered and well-tolerated by patients and have the advantage of not increasing the risk of metastasis.

## References

[1] Cormier JN, Askew RL, Mungovan KS, Xing Y, Ross MI, Armer JM (2010). Lymphedema beyond breast cancer: a systematic review and meta-analysis of cancer-related secondary lymphedema. Cancer.

[2] Mihara M, Hara H, Hayashi Y (2012). Pathological steps of cancer-related lymphedema: histological changes in the collecting lymphatic vessels after lymphadenectomy. PLoS One.

[3] Kwan ML, Darbinian J, Schmitz KH, Citron R, Partee P, Kutner SE, Kushi LH (2010). Risk factors for lymphedema in a prospective breast cancer survivorship study: the pathways study. Arch Surg.

[4] Wynn TA (2008). Cellular and molecular mechanisms of fibrosis. J Pathol.

[5] Baker A, Kim H, Semple JL, Dumont D, Shoichet M, Tobbia D, Johnston M (2010). Experimental assessment of pro-lymphangiogenic growth factors in the treatment of post-surgical lymphedema following lymphadenectomy. Breast Cancer Res.

[6] Hartiala P, Saarikko AM (2016). Lymphangiogenesis and lymphangiogenic growth factors. J Reconstr Microsurg.

[7] Joseph WJ, Aschen S, Ghanta S (2014). Sterile inflammation after lymph node transfer improves lymphatic function and regeneration. Plast Reconstr Surg.

[8] Kitchen G, Garrett MJ (1971). A trial of intra-lymphatic cyclophosphamide in patients with arm lymphoedema due to metastatic breast carcinoma. Clin Radiol.

[9] Kasseroller RG, Schrauzer GN (2000). Treatment of secondary lymphedema of the arm with physical decongestive therapy and sodium selenite: a review. Am J Ther.

[10] Micke O, Bruns F, Mucke R (2003). Selenium in the treatment of radiation-associated secondary lymphedema. Int J Radiat Oncol Biol Phys.

[11] Zimmermann T, Leonhardt H, Kersting S, Albrecht S, Range U, Eckelt U (2005). Reduction of postoperative lymphedema after oral tumor surgery with sodium selenite. Biol Trace Elem Res.

[12] Nakamura K, Radhakrishnan K, Wong YM, Rockson SG (2009). Anti-inflammatory pharmacotherapy with ketoprofen ameliorates experimental lymphatic vascular insufficiency in mice. PloS One.

[13] Gardenier JC, Kataru RP, Hespe GE (2017). Topical tacrolimus for the treatment of secondary lymphedema. Nat Commun.

[14] Rockson SG, Tian W, Jiang X (2018). Pilot studies demonstrate the potential benefits of antiinflammatory therapy in human lymphedema. JCI Insight.

[15] Appleyard CB, McCafferty DM, Tigley AW, Swain MG, Wallace JL (1996). Tumor necrosis factor mediation of NSAID-induced gastric damage: role of leukocyte adherence. Am J Physiol.

